# The prevalence of “novel” food allergens worldwide: a systematic review

**DOI:** 10.1186/2045-7022-5-S3-P9

**Published:** 2015-03-30

**Authors:** Harriet Moonesinghe, Sally Kilburn, Heather Mackenzie, Carina Venter, Kellyn Lee, Tara Dean

**Affiliations:** 1 University of Portsmouth, Portsmouth, UK

## Background

Much is known about the prevalence of common food allergens such as milk, egg, peanut, tree nut, fish and shellfish. However little is known of the true prevalence of other 'novel' food allergens.

## Aim

To use systematic review methodology to examine the worldwide prevalence of celery, lupin, mustard and sesame allergy.

## Method

Searches were conducted using two databases; Web of Science and PubMed. Initially 7333 studies were identified; 7145 were excluded at title/abstract screen and 99 at full text screen. There were 92 studies included in the review, 14 of which reported data on the novel allergens listed above.

## Results

Celery: four studies reported on the prevalence of celery allergy in Europe, which ranged between 2.8-11.1% based on sensitisation, and 5.5% for self-reported celery allergy. In terms of the rest of the world, only one study in Taiwan reported sensitisation rates of 1.8%.

Lupin: no population-based studies examined the prevalence of lupin allergy worldwide.

Mustard: only one study (conducted in France, Europe) examined mustard allergy finding self-reported prevalence of 3%.

Sesame: eight studies reported on the prevalence of sesame allergy in Europe, reporting a prevalence range of 0-1.5% for self-reported sesame allergy, 0-2.2% for sensitisation rates and 0.1-0.6% for challenge-proven allergy. There were four studies carried out in other regions of the world, with prevalence ranging from 0-0.2% self-reported, 0.2-1.6% sensitisation and 0.7% challenge-proven sesame allergy.

## Conclusion

There is surprisingly little data available on the prevalence of these novel food allergens despite the fact that they appear in the top 14 food allergens listed by the EU. Furthermore, the gold-standard of diagnosis, food challenges, were only adopted in two studies looking at sesame allergy and these were conducted in children.

